# Air Puff System Fundamentals for Reproducible Eyeblink Conditioning Research

**DOI:** 10.3390/mps2010014

**Published:** 2019-02-02

**Authors:** Frederick Reitz

**Affiliations:** University of Washington Autism Center, Center on Human Development and Disability, University of Washington, Box 357920, Seattle, WA 98195-7920, USA; freitz@uw.edu

**Keywords:** eyeblink conditioning, air puff, system design, characterization

## Abstract

Air puff systems are at once trivially straightforward and dauntingly complex. On the one hand, they are little but a pressure source, valve, and tube connected together. On the other, the air passing through them is a compressible medium, expanding approximately adiabatically while travelling at high velocity through a compliant tube, and exiting as a turbulent jet with velocity peak and profile varying non-linearly in its near-field. This complexity puts precise mathematical prediction of puff properties out of reach of most labs. There are, however, a number of phenomena fundamental to air puff system design that are worth understanding to a first order of approximation, or at least qualitatively. Using a simplified, “electronic–hydraulic analogy” model, this paper discusses these phenomena in just enough depth for the reader to confidently specify parts for an air puff delivery system, to measure its key parameters, and/or to describe a given system unambiguously in publications, thus maximizing reproducibility.

## 1. Introduction

In classical, or Pavlovian, conditioning, two stimuli are presented together, one of which (the “unconditioned stimulus”, or “US”) elicits a particular automatic response (the “unconditioned response”, or “UR”), while the other (the “conditioned stimulus”, or “CS”) does not. After enough exposure, the subject’s neural systems associate the US and CS with each other such that the automatic response begins to occur in response to the previously unrelated stimulus. For example, Pavlov’s famous dog learned to salivate at the sound of a bell, even in the absence of co-presented food (the “conditioned response”, or “CR”) [1].

The neural pathways underlying this association vary with the stimulus; fear conditioning requires the amygdala [2], the cerebellum is central to eyeblink conditioning [3,4,5,6], and the prefrontal cortex is called into play by introducing a delay between CS and US (i.e., “trace” eyeblink conditioning; [7]). Classical conditioning experiments have been used to study myriad conditions, including fetal alcohol syndrome, Down syndrome, fragile X syndrome, attention deficit/hyperactivity disorder, dyslexia, specific language impairment, schizophrenia, and autism [8].

Animals have many reflexes that can be conditioned in this way. Blinking to protect the eye from a sudden physical stimulus is relatively straightforward to elicit and measure, and can be triggered safely via a puff of air directed at the eye [6,8]. When paired with a CS, a corresponding CR may be elicited, albeit after hundreds of such trials [9,10]. Eyeblink conditioning apparati typically consist of a source of pressurized air, a tube routing this air to the eye, and a solenoid valve that can be triggered as necessary to deliver puffs. The simplicity of this arrangement is at once refreshing and deceptive; it is the rare piece of scientific equipment that can be understood at a glance, though as will become apparent shortly, this surface understanding can fall far short of that needed for scientific reproducibility, and two systems described by the same pressure can produce puffs of radically differing intensity.

If a subject complains that puffs are too strong, or an animal fails to blink, may the experimenter not adjust the pressure as needed with impunity, concerned only that it be enough to elicit a response? If UR amplitude varies between subjects, may this be assumed independent of trivial hardware tweaks that go unrecorded? A number of studies have found a strong dependence of conditioning degree and/or rate on the intensity of air puffs used on both humans [11,12] and rabbits [13,14,15], calling this into question. US-alone trials provide a measure of the degree of UR to the stimulus used, allowing informative comparison to the CR, though even this relationship can depend upon US intensity, which facilitates or diminishes the UR with conditioning accordingly [16], and points up the importance of explicitly characterizing the puff itself.

## 2. Materials and Methods

### 2.1. Automated Flow Rate Measurements

**Figure 1:**
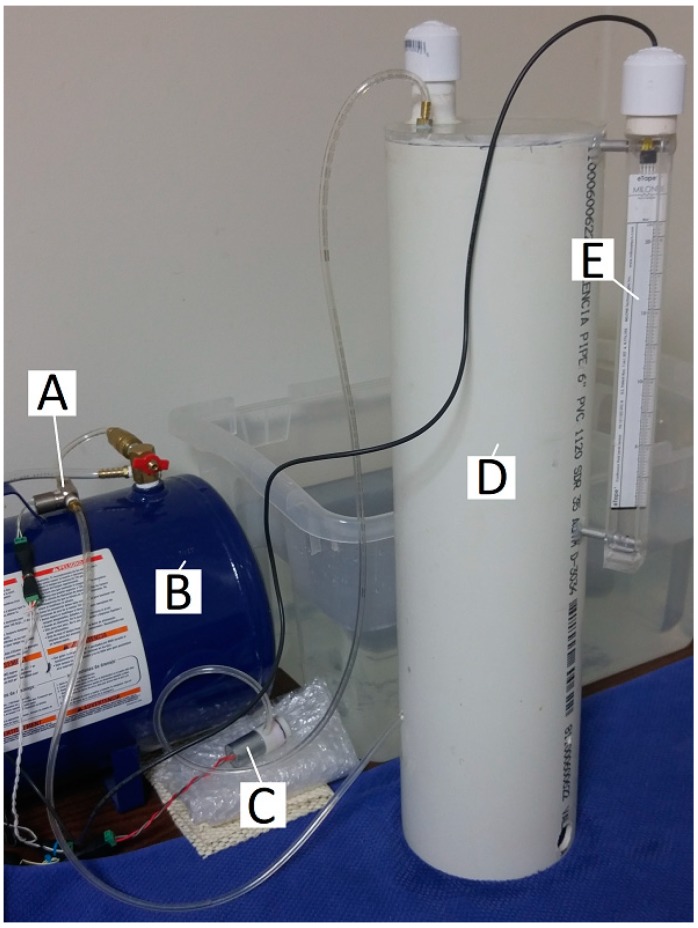
View of the system used herein, showing (**A**) solenoid valve, (**B**) air-carry tank, (**C**) vacuum pump, (**D**) air-trap chamber (removed from the water reservoir for visibility), (**E**) water depth transducer.

To facilitate the large number of measurements required for this study, an automated flow-measurement system was constructed as shown in Figure 1. The system is monitored and controlled with custom LabVIEW software via a myDAQ multi-function I/O device (National Instruments, Austin, TX, USA).

**Figure 2:**
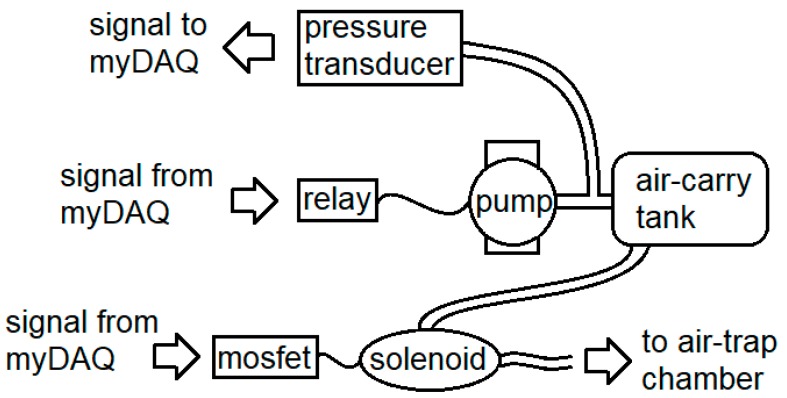
The air-handling subsystem: A digital signal activates a relay that powers on an air compressor (the “pump”), pressurizing an air-carry tank monitored via a pressure transducer. Puffs are then released by another digital signal switching on the solenoid valve with a MOSFET.

The air control portion of the system is schematized in Figure 2. A digital signal from the myDAQ controls a relay that switches on a 1/5-HP airbrush compressor, pressurizing a 5-gallon air-carry tank. The software monitors the status of this process via a pressure transducer (MPXV5100, Motorola Solutions, Inc., Chicago, IL, USA) and stops at the desired pressure. Another digital signal from the myDAQ I/O device switches on the solenoid valve via a MOSFET, releasing a puff of air from the tank.

**Figure 3:**
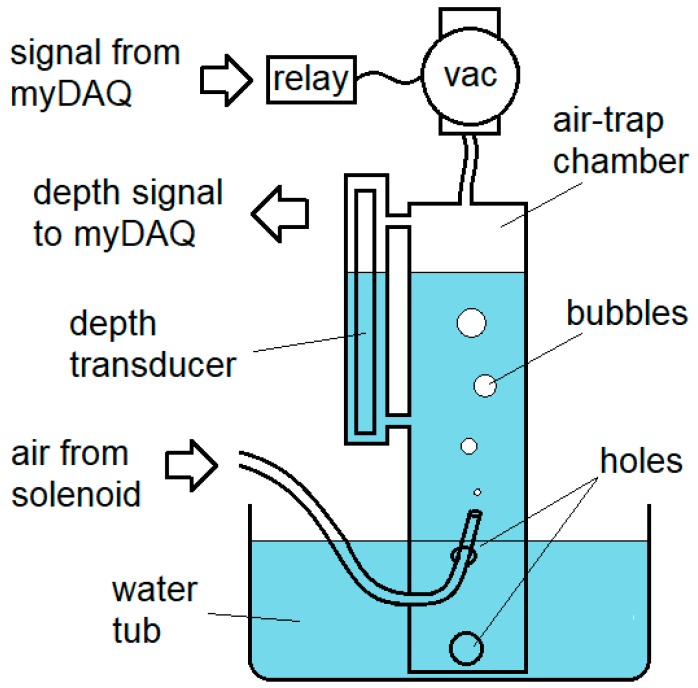
The volume-measurement subsystem: Air from the solenoid and tubing, under test, enters an air-trap chamber, displacing water therein, as measured with a depth transducer. The system is reset with another digital signal activating a vacuum pump (“vac”) via a relay, thereby drawing water back up into the chamber.

The puff volume measurement portion of the system is schematized in Figure 3. Before a measurement, a digital signal activates, via a relay, a vacuum pump, drawing water up into the air-trap chamber via holes drilled in the side of the chamber from a 64-quart tub of water, in which it stands. The level of the water in the air-trap chamber is monitored via depth transducer, (8″ eTape, Milone Technologies, Sewell, NJ), and the vacuum is switched off when the water level nears the top of the transducer’s range. The end of the tubing, under test, extends into the chamber to approximately the level of the water in the surrounding tub, and air is puffed into the air-trap chamber until the water level drops to near the bottom of the depth transducer’s range.

Before and after the puff, the software waits for the variance in the depth signal from water waves to die down completely, and notes the water depth. By timing the signal provided to the solenoid, the software may then calculate a volume difference per elapsed time, and thus a flow rate. Sources of measurement error are considered in Appendix A.

### 2.2. Intra-Tube Pressure Dynamics

**Figure 4:**
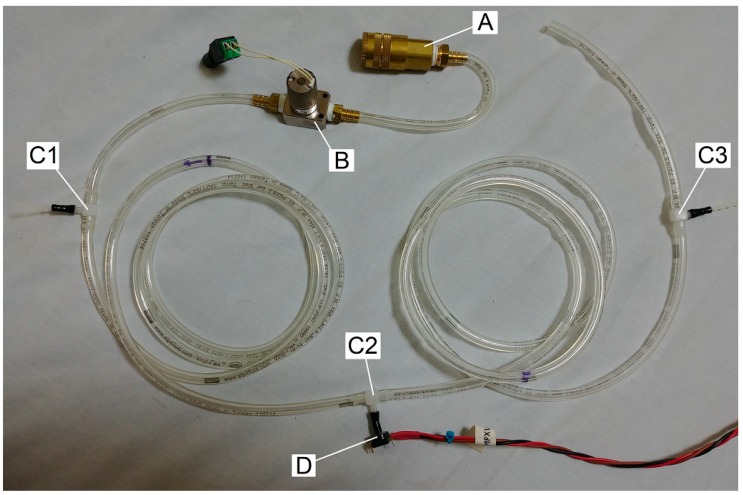
Air comes from the pressurized tank (not shown) through a quick connector (**A**), into tubing into which have been spliced a solenoid valve (**B**) and three 1/8″ T-connectors (**C1**, **C2**, and **C3**), creating ports at which the pressure inside the tube may be monitored by inserting a pressure transducer (**D**). Ports not in use are capped.

**Figure 5:**
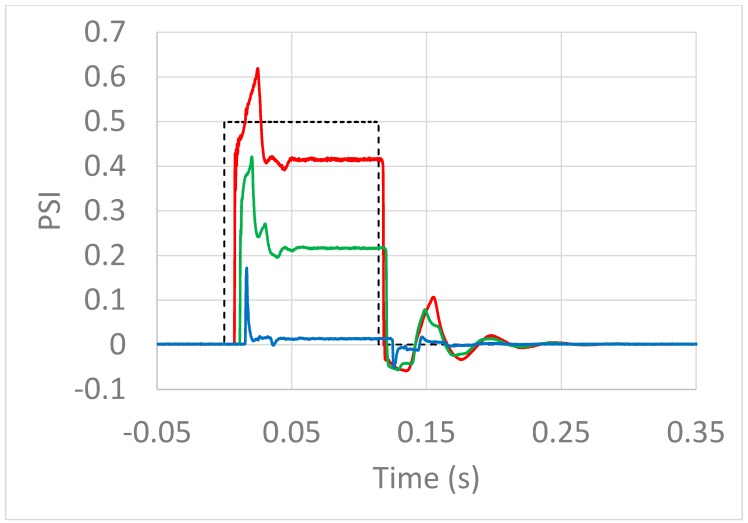
A “5 PSI” pressure pulse measured at 6″ after the solenoid valve (red), halfway down the 10′ tube (green), and 6″ before the tube exit (blue). The dotted line shows the timing of the solenoid control signal.

Using a length of tubing with T-connectors as shown in Figure 4, we can observe the pressure within the tube during puffs, yielding the traces shown in Figure 5.

Air comes from the pressurized tank (not shown in Figure 4) through a quick connector (A), into 1/8” i.d. tubing into which have been spliced a solenoid valve (B; an EM-2-12, Clippard, Cincinnati, OH), and three 1/8″ T-connectors spliced into the tubing (C1, C2, and C3; capped off when not in use), at which points the pressure inside the tube may be monitored by inserting a pressure transducer (D; MPXV5010, NXP, Eindhoven, The Netherlands).

The pressure and solenoid activation signals were read by additional custom LabVIEW software and the myDAQ multi-function I/O device at 10,000 samples per second.

## 3. Results

### 3.1. What’s Really Happening within the Tube

Source PSI (i.e., source pressure in pounds per square inch, where 1 PSI ~ 0.07 bar) is typically reported as the primary (and often only) descriptor of puffs used in a study, but closer inspection reveals that this falls far short of describing the puff.

Figure 5 shows the pressure pulse from a 5 PSI source into 10′ of 1/8″ i.d. tubing over time as measured at 3 different positions: (1) 6″ after the solenoid (red), (2) halfway down the tube (green), and (3) 6” before the tube exit (blue). From this we can make four observations: (1) At none of these measurement locations does the pressure reach 5 PSI, or even 1 PSI; (2) the pressure pulse further attenuates with distance along the tube, until (3) near the exit of the tube, the deviation from ambient pressure is near zero, and (4) the pressure in the tube oscillates for almost 0.1 s before reaching a steady state.

The oscillation can be explained by analogy to a wind instrument; when air flow is abruptly started or stopped at one end of a tube, the air in the tube reverberates at a frequency dictated by the length of the tube [17].

We explore observations 1, 2, and 3 in the next section.

### 3.2. The “Electronic–Hydraulic Analogy” Model of Pressure, Flow, and Resistance to Flow

As the air pathways through a vacuum cleaner become clogged, the vacuum cleaner progressively loses suction. Its motorized pump continues to strain as hard as ever, but the pressure difference that it creates between the inside of the cleaner and the ambient atmospheric pressure outside the cleaner results in little actual movement of air, as the flow is resisted by obstruction in the pathways.

Likewise, a car tire inflated to 30 PSI may go flat via a “blowout” that sounds like a gunshot and leaves the tire in shreds, or via a leak so subtle as to require the application of soapy water to detect. These are both “30-PSI puffs”, though at opposite ends of the resistance spectrum; in the latter case, the flow encounters near-total resistance from the almost-completely-intact tire, while in the former, a large tear allows the air to rush out unimpeded, and thus explosively.

In an air puff system, though a solenoid and narrow tube may open a path from, say, a reservoir of 5 PSI compressed air on one end to open atmosphere at the other, the reservoir is (hopefully) not depleted of pressure in such a dramatic instant; it takes time for the air to escape through the system, because the restricted aperture of the solenoid and tubing resists this flow.

Neglecting all non-linear effects (of which, admittedly, there are many), the rate of air flow *F* through a given channel will be approximately

*F* ≈ *P*/*R*, (1)

where *P* is the difference in pressure between the inlet and outlet, and the resistance to flow, *R*, in this simplified model, is a constant associated with the channel, dictated predominantly by its geometry.

This equation is closely analogous to Ohm’s Law, describing current flow in electrical circuits, with pressure corresponding to voltage, flow to amperage, and flow resistance to ohms [18,19].

**Figure 6:**
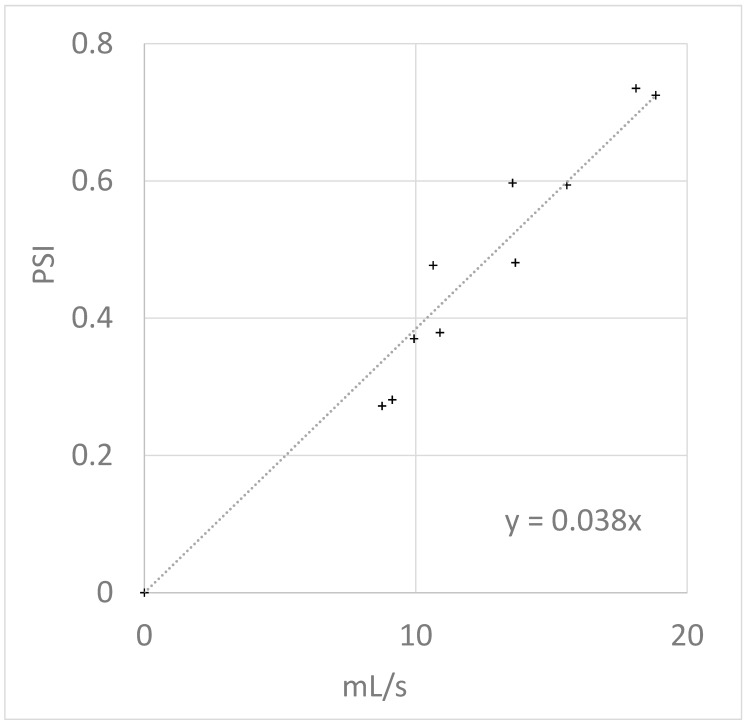
Required pressure differential as a function of flow rate.

Figure 6 shows measurements of an actual solenoid valve (2V025-06 solenoid (Uxcell, New Territories, Hong Kong, China) and 9″ of 1/16″ i.d. tubing) demonstrating reasonable agreement with this theory. The plot has a slope, and thus resistance constant, of ~0.038 PSI-s/mL.

In this model, as with Ohm’s Law, the resistance of an entire air puff system is simply the sum of its parts, so

*R*_total_ ≈ *R*_source_ + *R*_solenoid_ + *R*_tubing_, (2)

where *R*_source_ is the resistance of any fittings and tubing between the pressure source and the solenoid valve, *R*_solenoid_ is that of the solenoid itself and any attached fittings, *R*_tubing_ is that of tubing after the solenoid, and *R*_total_ is the resistance of the entire system.

The linearity of this model suggests a few rules of thumb:

(a) If the resistance of 1 foot of tubing is *R*_1foot_, the resistance of 10 feet of tubing will be approximately the sum of 10 such resistances, or 10*R*_1foot_;
(b) Doubling the resistance at a given pressure should approximately halve the flow rate;
(c) Doubling the pressure should approximately double the flow rate.

**Figure 7:**
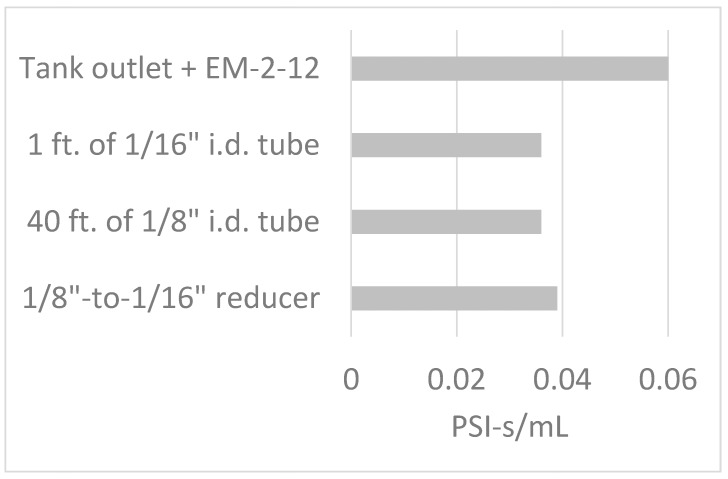
Resistance measurements of typical air puff system components.

**Figure 8:**
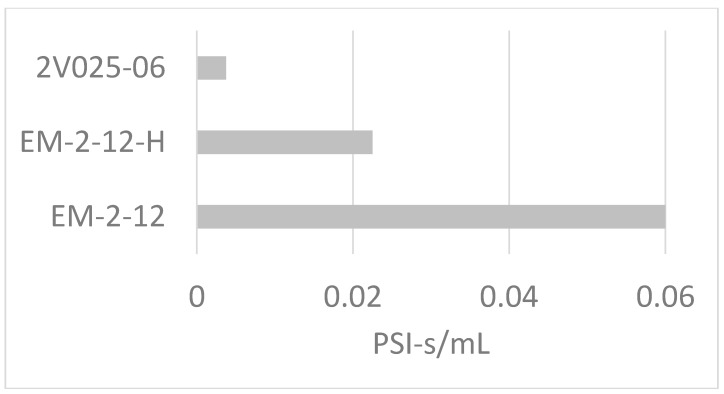
Resistance measurements of different solenoid models: The 2V025-06 is designed for high flow rate without regard to audibility. The EM-2-12-H and EM-2-12 are designed for quiet operation at low (<25 PSI) and high (up to 105 PSI) pressures, respectively.

Figure 7 and Figure 8 show the measured resistances of typical components of an air puff system. From Figure 7 we can see that:

(a) A single coupler such as a plastic reducing adapter is comparably resistive to a foot of 1/16″ i.d. tubing;
(b) A single foot of 1/16″ i.d. tubing is comparably resistive to 40 feet of 1/8″ i.d. tubing;
(c) The solenoid and tank outlet contribute to the resistance significantly in and of themselves.

Meanwhile, Figure 8 shows that:

(d) Resistance among solenoids that appear similar can vary by more than one order of magnitude, so choice of solenoid can greatly influence exactly what PSI will be required to achieve a desired strength of puff. Note: the 2V025-06 is included as a high-flow-rate example only; it is not designed for quiet operation.

We are now in a position to explain the remaining observations from Figure 5.

*Observation 1: At none of these measurement locations does the pressure reach 5 PSI, or even 1 PSI.*

The resistance of the solenoid used was greater than the subsequent tubing, such that the pressure drop across the solenoid was proportionally large, and most of the source pressure was “spent” traversing it, such that the pressure measured even almost immediately after it was <1 PSI.

*Observation 2: The pressure pulse further attenuates with distance along the tube.*

The pressure drop across a length of tubing is approximately proportional to the resistance of that length, such that half of the pressure drop across the tubing will occur over the first half of the tubing.

*Observation 3: At the exit of the tube, the deviation from ambient pressure is near zero.*

The resistance between the tubing exit and the surrounding atmosphere is near zero, such that the proportional amount of pressure drop occurring beyond the end of the tube is near zero. Like a ground wire in an electric circuit, the end of the tube is connected to a vast reservoir held at a potential defined as zero (in this case, zero gauge pressure).

### 3.3. “Puff” Geometry

Now let us consider an air puff system with a 10 mL/sec flow rate through 1/16″ i.d. tubing. This corresponds to (a) a Reynolds number of approximately 500 (see Appendix A), and (b) an average air velocity of approximately 5 m/s, or 50 cm per 0.1 s, the duration of a typical puff.

In the absence of the experimental subject, the “puff” would quickly come to resemble what fluid dynamicists would call an “axisymmetric turbulent jet”, that is, a sustained flow, from a round orifice, that adopts a predictable geometry [20].

**Figure 9:**
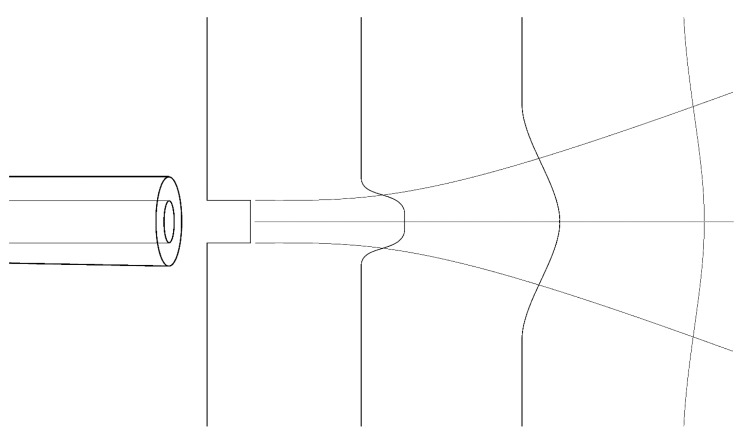
Qualitative profile of a puff.

Such jets have a number of properties, illustrated qualitatively in Figure 9:

1. There is a near-field region in which the flow remains somewhat collimated, and square in velocity profile, for a short distance (~10 times the aperture diameter).
2. The flow then diverges with a half-angle of approximately 12°, gradually adopting a Gaussian velocity profile.
3. The peak (axial) velocity drops off monotonically toward zero with distance from the aperture as the velocity profile broadens laterally.

From this we see that:

- The flow profile shape, half-width, and peak velocity all vary with distance;
- This variation is particularly interesting at precisely the separation distances typically chosen by experimenters (i.e., “close to the eye”, where the flow is transitioning from the near- to far-field region);
- Where this interesting transition happens depends upon the diameter of the exit orifice of the system.

Does air with a square velocity profile feel different to one’s eye than a Gaussian profile? Does a broadly-distributed jet of a given momentum feel different from a narrowly-focused one? Is a puff of high peak velocity perceived as more intensely startling than a puff with lower peak velocity but comparable total momentum? And exactly how does placing the subject within this flow alter its dynamics?

Unless these questions may be answered or dismissed confidently, I urge experimenters to include note of the tubing-to-eye separation and tubing exit diameter in their reports.

## 4. Discussion

### 4.1. What Flow Rate Should One Choose?

Given that one can choose parts as needed to arrive at a desired flow rate, what flow rate is most desirable?

As it has not, to date, been the norm to report air puff system flow rates, and as puffs may need to be adjusted to suit the respective sensitivities of differing subjects, it is exceedingly difficult to say definitively what flow rate is preferable, or even typical, except that it should be not too weak and not too strong.

Still, I can offer an order-of-magnitude attempt at quantification via (A) my personal experience as a research engineer who has constructed several such systems and (B) measuring that of an off-the-shelf system with default settings as a benchmark.

Regarding (A), my subjective perceptions are that:

▪ 100 mL/s is too strong by far;
▪ 10 mL/s is rather strong but not obviously unacceptable;
▪ 1 mL/s is perceptible but subtle.

So, in this author’s hands, the answer is “on the order of 1 to 10 mL/s”.

For (B), I directly measured the flow rate of a commercially-available eyeblink conditioning system (San Diego Instruments, San Diego, CA, USA) as shipped to be 7 mL/s, consistent with the above impressions.

### 4.2. Measuring Flow Rate at Your Workbench

The precise measurement of the flow rate of air through a system is made non-trivial by a number of factors, including the compressibility of air, increased density due to temperature drop with expansion, and perturbation of the system by the measurement itself.

**Figure 10:**
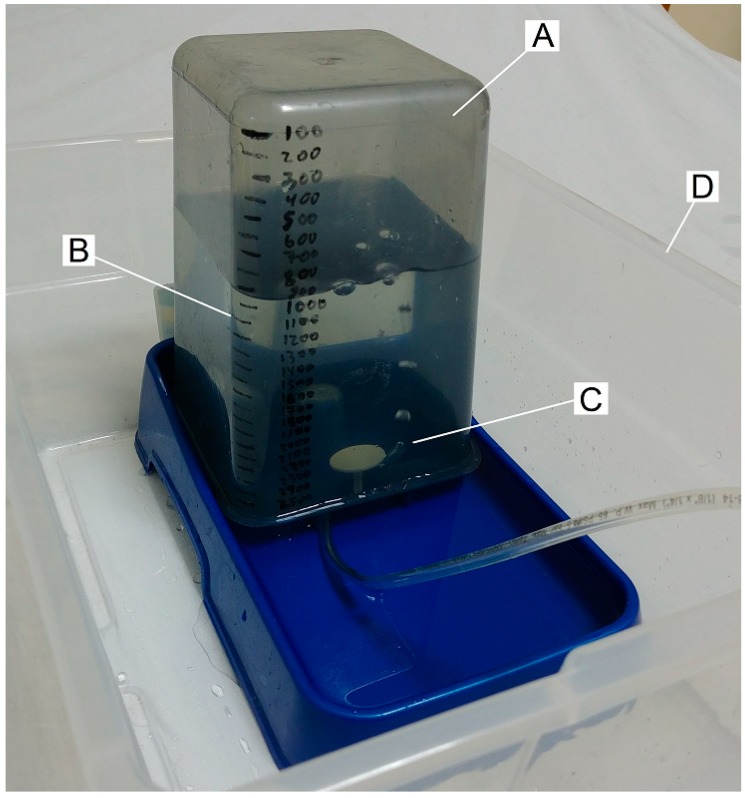
A conventional pet water dispenser (**A**) is volume-calibrated by filling its reservoir 100 mL at a time, and marking the levels (**B**) with a marker. The end of the tube under test is inserted up into the dispenser, blowing bubbles that are captured by the reservoir (**C**). By noting the water level before and after puffs of known duration, an approximate flow rate measurement is obtained. The measurement is performed within a larger tub (**D**) to contain spills.

Again, however, an approximate measurement can be easily made. Figure 10 shows a simple system for doing so.

If the outlet of the tube is approximately level with the surface of the water in the tray surrounding the reservoir, the hydrostatic pressure at the outlet will be that of the ambient air, thus minimizing perturbation of the system by application of back pressure. The level of the air–water interface within the reservoir will affect the pressure of the contained air, thus affecting volume (though negligibly, for this purpose), as the hydrostatic pressure of a 6″ water head is only ~0.2 PSI, resulting in less than a 2% difference in volume.

After a few seconds of air delivery, via a sustained puff or many brief puffs of known duration, a volume difference large enough to measure accurately can be thus determined, and approximate flow rate calculated.

## 5. Conclusions

To best enable others to reproduce a given experiment and/or meaningfully compare one system with another, I urge that the materials and methods section of an eyeblink conditioning paper include, in addition to source PSI, the following checklist:

1. The make and model of the solenoid or off-the-shelf system used;
2. the length and inner diameter of the tubing used;
3. the exit aperture diameter, if different from that of the tubing;
4. the tubing-eye separation distance.

This much will allow other experimenters, in principle at least, to estimate the resulting flow rate of the system and air jet geometry as it impacts the eye.

If possible, I also recommend including:

5. A direct measurement of the flow rate of the system, as the experimenter, with system in hand, is uniquely positioned to best determine this number.

## Appendix A. Error Analysis

### Appendix A.1. Adiabatic Expansion and Subsequent Temperature Equilibration

The air in a puff is expected to drop in temperature as it expands approximately adiabatically upon release from the storage tank, hence *P*^1-γ^*T*^γ^ and *PV*^γ^ each remain approximately constant, where *P* is pressure, *V* is volume, *T* is temperature in Kelvin, and γ is the heat capacity ratio of the air (approximately 1.4 for dry air near room temperature). The volume of this cool air will then increase somewhat upon re-equilibration with room temperature. As my level measurement appeared to be stable, within noise, over prolonged equilibration times, I conclude that this effect is smaller than the precision of my automated system.

### Appendix A.2. Varying Height within Water Column

If the tubing outlet is placed above or below the level of the water in the reservoir, the pressure differential across the system will vary by approximately 0.5 PSI per foot of water head. Care was taken to match these levels to within approximately an inch, corresponding to ~0.04 PSI of error.

### Appendix A.3. Reynolds Number

The Reynolds number Re of a flow is given by

*Re* = *ρvL*/*μ*, (A1)

where *ρ* is the density of the fluid, *v* is its velocity, *L* is the characteristic dimension of the system (i.d. in the case of a pipe), and *μ* is the dynamic viscosity of the fluid. This number is essentially a ratio of inertial to viscous forces, which is a key determinant of the nature of the flow, in particular, whether it will be laminar or turbulent. In a tube, this transition ordinarily begins at a Reynolds number on the order of 2000, corresponding a flow rate of some tens of mL per second for commonly-used tubing diameters; hence, above, but not far from, the regime of operation of an air puff system. I did consistently observe something of a “kink” in my pressure/flow-rate curves when extended to high flow rates, likely corresponding to this transition, though as I deemed these rates beyond those useful to eyeblink conditioning, I did not attempt to account for this deviation from linearity in the simplified model discussed herein.

### Appendix A.4. Calibration

Flow rate was determined herein by measuring the difference in height of a water column accumulated over several seconds, with computer-controlled solenoid switching. The displaced volume was calculated by assuming that the inner diameter of the air-trap chamber was exactly 6 inches; that the solenoid opening and closing were instant, or at least symmetrical in profile, such that any delays canceled out; and that the depth transducer was perfectly accurate. The inner diameter of the chamber was verified by direct measurement to be within ~1/32 of an inch, potentially contributing ~1% volume calculation error. The switching times for the solenoids used were on the order of ~10 ms, and held open for varying durations ranging from ~1 to 10 s, hence also contributing as much as 1% error. The depth gauge was calibrated by fitting its readings to the ruler-measured depth of water via a 3rd-order polynomial, resulting in a calibration curve observed to be reproducible within ~0.3″ of depth. To minimize the impact of this uncertainty, measurements were sustained until approximately 6 inches of water had been displaced, resulting in an error margin of ~5%.

### Appendix A.5. Steady-State Approximation of Transient Puffs

As noted, the pressure in the tube oscillates about its steady-state value for a significant portion of the duration of a typical puff. I have assumed herein that this phenomenon contributes negligibly, or at least in a way that averages to zero, to the order-of-magnitude resistance measurements obtained via prolonged (several second) solenoid activations. In extreme cases, however (especially brief puffs through especially long tubing), I observed average intra-tube pressures during the pulse as much as 18% higher than its steady-state value, suggesting that more exacting measurements of flow rate might be obtained by averaging together many brief puffs rather than a single, sustained one.
